# Don’t Throw the ‘Bio’ out of the Bio-Psycho-Social Model: Editorial for Spine Rehabilitation in 2022 and Beyond

**DOI:** 10.3390/jcm12175602

**Published:** 2023-08-28

**Authors:** Deed E. Harrison, Paul A. Oakley, Ibrahim M. Moustafa

**Affiliations:** 1 CBP Nonprofit (a Spine Research Foundation), Eagle, ID 83616, USA; 2 Independent Researcher, Newmarket, ON L3Y 8Y8, Canada; docoakley.icc@gmail.com; 3 Kinesiology and Health Science, York University, Toronto, ON M3J 1P3, Canada; 4 Neuromusculoskeletal Rehabilitation Research Group, RIMHS–Research Institute of Medical and Health Sciences, University of Sharjah, Sharjah 27272, United Arab Emirates; iabuamr@sharjah.ac.ae; 5 Department of Physiotherapy, College of Health Sciences, University of Sharjah, Sharjah 27272, United Arab Emirates

## 1. Introduction

Spinal injuries, disorders and disabilities are among the leading causes for work loss, suffering, and health care expenditures throughout the industrialized world [1,2,3,4,5,6]. The psycho-social and economic impact of general and specific spine disorders demands continued research into the most effective types of preventative and interventional treatment strategies. Specifically, low-back-pain (LBP)- and neck-pain-related disorders are the 1st and 4th leading causes of work loss and disability in the world [1,2,3,4,5,6]. Though billions are spent annually in experimental, epidemiology, and interventional strategies, precise treatment regimens aimed towards improving, resolving, and preventing these spinal disorders are highly varied and have limited and/or only short-term efficacy [1,2,3,4,5,6]. Thus, spinal disorders and related disabilities remain a high priority research avenue within the health sciences; in particular, there is an urgent need to increase the knowledge related to the manual rehabilitation disciplines [5,6].

Pain and disability with a spinal origin have several proposed psycho-social [1,2,3,4,5,6,7,8,9] and biomechanical contributing factors [10,11] which has given rise to the well-known ‘bio-psycho-social’ model of understanding injury mechanisms leading to the development of chronic pain and disabilities. Problematically, in recent decades, many authors have begun to minimize the ‘bio’ (tissue injury, damage, anatomical disorder, etc.) component of the problem, thus favoring the ‘psycho-social’ aspects such as catastrophizing, fear/anxiety and avoidance behavior components in the development of chronic pain in the patient [1,2,3,4,5,6,7,8,9], as some authors are quite adamant that the ‘tissue injury’ component plays a rather limited role [6]. It can be argued, though, that the lack of appreciation for the tissue component of spine pain/disorders is shortsighted, based on an incomplete review of recent systematic reviews, and based on limitations with early analytical methods, whereas today’s technology and more detailed investigations have identified a significant role for the tissue component as contributing to the presence and development of chronic spine pain and disability [12,13,14,15]. Furthermore, proponents of the stronger role that the ‘psycho-social’ part of the equation plays in spine conditions often fail to acknowledge that recent systematic literature reviews with meta-analysis have identified a clear controversy regarding the quality and true impact that fear-avoidance, pain-catastrophizing (PC), and ‘psycho-social’ model elements play in individuals with chronic musculoskeletal pain (CMP) disorders [7,8,9]; for example, the following has been stated: “*Despite the very low quality of the available evidence, the general consistency of the findings highlights the potential role that PC may play in delaying recovery from CMP. Research that uses higher quality study designs and procedures would allow for more definitive conclusions regarding the impact of PC on pain and function*.” [7]. The current authors of this Editorial offer this perspective for context and not to dismiss the role that the psycho-social component plays in initiating and developing chronic spine related disorders.

It is often understood but understated that the ‘bio’ component in the ‘bio-psycho-social’ model also stands for biomechanics (not just biology) either segmentally or globally of the whole spine–body system [10,11]. While the mechanical causes of musculo-skeletal pain are not completely understood, they are thought to be linked to the interconnected functions of anatomical components (soft and hard tissues) of the spine where injury and pain can be caused by any incident that alters joint mechanics (kinematics, kinetics, alignment), tissue integrity, and muscle function via alterations and increases in general loading and load sharing of the various tissues [10,11]. Of interest, several authors have attempted to completely dismiss or minimize the role that biomechanics (alignment and loading) plays in the onset and development of musculoskeletal disorders [16,17,18,19,20]. For example, in a systematic review, it was concluded that “*Evidence from epidemiological studies does not support an association between sagittal spinal curves and health including spinal pain*.”[16]. Complicating the matter, in each of the reviews that proposed a minimization of the role that biomechanics (alignment and loading) plays in chronic spine disorders [16,17,18,19,20], serious flaws in the study design and literature reviews were identified [19,20,21,22,23,24] highlighting the controversy and confusing the situation further.

Importantly, in the past two decades, the role that biomechanics of the sagittal plane alignment of the spine and three-dimensional posture has on human performance, health, pain, disability, and diseases has been a primary research focus among spine surgical and rehabilitation specialists across the scientific literature [25,26,27,28,29,30,31,32,33,34,35,36,37]. It has been quite extensively demonstrated that sagittal plane alignment and biomechanics of the lumbo-pelvic [25,26,27,28,29,30,31,32], thoracic hyper-kyphosis [33,34,35], and cervical [36,37] spines have clear impacts on human health and well-being, musculoskeletal disorders, and chronic pain disorders. Limits of normality for a variety of sagittal spine alignment parameters have been documented, providing chiropractors, physical therapists, surgeons, and other spine specialists with standardized goals to compare patients to in both pre- and post-treatment decision-making strategies [25,26,27,28,29,30,31,32,33,34,35,36,37,38,39,40]. Furthermore, conservative interventional methods have been developed and tested for their effects on improving altered sagittal plane alignment and preliminary and promising results have been found for a multi-modal program including lumbar extension traction (LET) [38], cervical extension traction (CET) [39], thoracic extension traction (TET) [40], bracing for thoracic hyper-kyphosis [41], and various specific exercise regimens for thoracic-kyphosis [41,42]. Problematically, some authors continue to ignore the evidence for these new types of sagittal plane curve-inducing (LET and CET) and curve-reducing (TET) traction methods and spinal bracing and their role in improving the sagittal plane alignment of the spine and improving chronic musculoskeletal disorders [4].

## 2. Purposes of Special Issue on Spine Rehabilitation

All too familiar are approaches to spine care involving functional rehabilitation programs including exercises for strength gains, range of motion increases, generalized stretching and strengthening procedures, massage, and soft tissue manipulation techniques, as well as physiotherapeutic modalities such as ultrasound and muscle stimulation, etc. An alternative to the traditional and popular functional approaches is a structural rehabilitation approach. Structural rehabilitation involves some aspects of functional rehabilitation methods but focuses on unique types of posture and spine correction methods for the primary purpose to realign and ‘over-correct’ the spine and altered postures [43].

**Figure 1:**
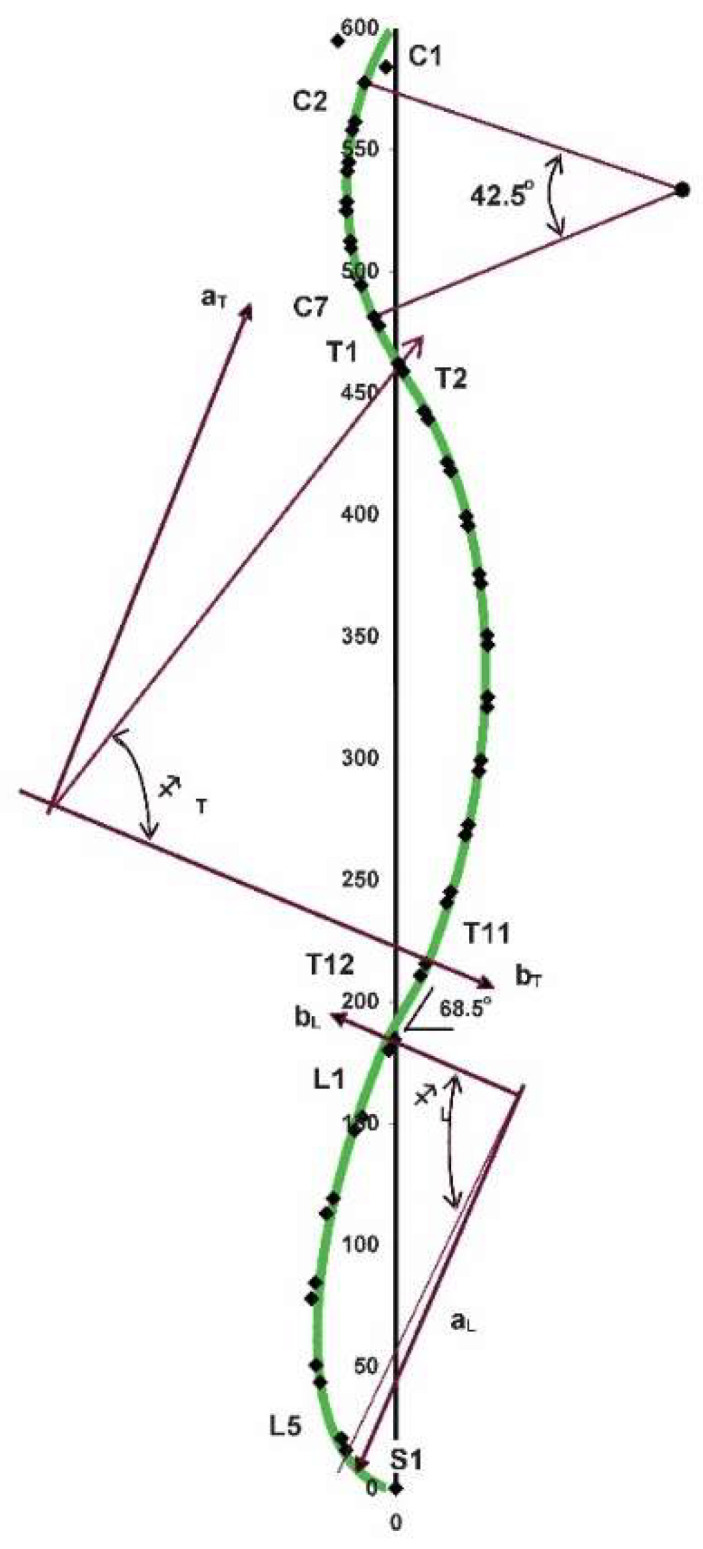
This diagram is the CBP^®^ Full spine Normal Model. It documents the proper path of the spine from a side view. Ideally, the back of your vertebra should align along this mathematical model. It is composed of specific ellipses as shown in the following regions on the left: • C1-T1: cervical (neck) • T1-T12: thoracic (rib cage) • T12-S1 lumbar (low back). The ideal spine has near perfect vertical balance of the upper- and lower-most vertebra for each of these three spinal regions. Each region has points of inflection—the mathematical term for change in direction from concavity to convexity with which to compare your six spinal X-rays against. Along the entire spine, each vertebra has a graphed mathematical point to correspond to. Such a spinal analysis helps determine proper (or improper) posture and alignment and how much correction may be required.

**Figure 2:**
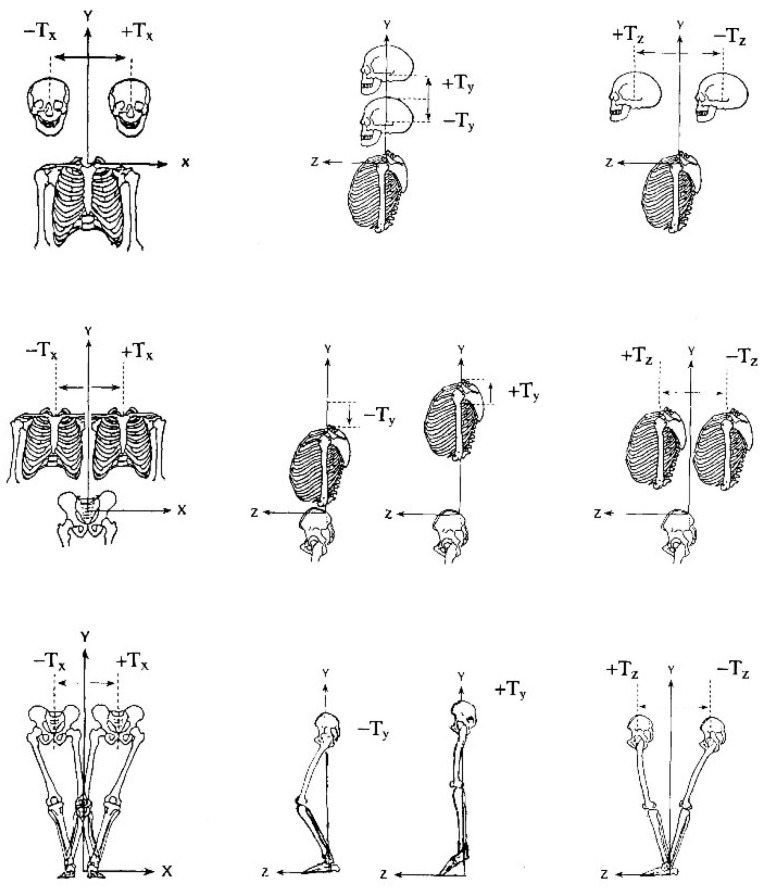
**Translational Components of Abnormal Body Postures.** In each region (head, ribcage, and pelvis), six distinct translation displacements are shown with “engineering” lines. Thus, 18 postural abnormalities as single postures are shown.

**Figure 3:**
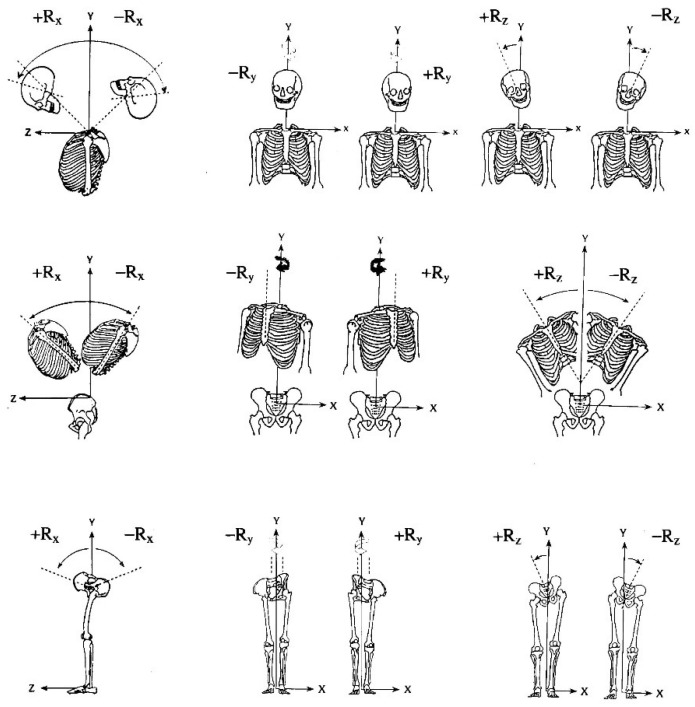
**Rotational Components of Abnormal Body Postures.** In each region (head, ribcage, and pelvis), six distinct rotation displacements are shown with “engineering” lines. Thus, 18 postural abnormalities as single postures are shown.

Although spinal bracing and postural exercise techniques have shown preliminary evidence for providing structural spine and posture realignment [41,42], one evolved technique that has laid a substantial foundation towards the structural approach to spine care is the Chiropractic Biophysics^®^ technique group [43]. From the mid-1990s to the mid-2000s, the Harrison research team performed a series of spine modeling studies of the sagittal spinal curves (Figure 1) [43]. This has formed the foundational spinal model to which patient comparison can be made for initial assessment of alignment abnormality and follow-up assessment to monitor treatment effects. Further, elaborate assessment and corrective treatment methods are based on the fundamental assessment of posture in terms of translations and rotations of the separate body segments in relation to each other (Figure 2 and Figure 3) [43].

Beginning in approximately 2010, Moustafa and colleagues (teaming up with Harrison and later Oakley) spearheaded the fundamental missing randomized controlled trials (RCTs) seeking to understand the efficacy and clinical utility of CET and LET methods [38,39,44,45]. These RCTs demonstrated that patients with cervical, thoracic, and lumbo-pelvic sagittal plane abnormality-related symptoms receiving spine correction via CET and LET methods achieved greater long-term health outcomes (pain, disability, mobility, etc.) versus patients who only received conventional functional based treatments that do not consistently improve spinal alignment [38,39,44,45]. Though today there are reliable and predictable means to restore the natural curvatures of the spine and improve sagittal balance and generalized posture alignment [38,39,40,41,42], the evidence is still preliminary and there are many areas for further research including the need for randomized trials on TET methods, an understanding of which sub-groups of populations with spine disorders might benefit the most, what is the ideal dose–response of treatment frequency and durations versus outcomes for different patient populations, and many other areas. Furthermore, more information from better quality case–control designs and cohort populations are needed to identify what type of effects (if any) that specific spine displacements have on musculoskeletal function, neurophysiology, and performance; in other words, more than just pain and disability outcome measures must be looked at and understood for a comprehensive understanding of the impact that altered spine/posture alignment has on spine related disorders and in improving human health and well-being. Additionally, there is a lack of information on non-sagittal spine displacements, and how these spine and posture displacements impact human health and disease needs to be comprehensively investigated. Finally, the economic impact, health benefits, and generalized awareness of full spine displacements and the newer ‘structural rehabilitation’ spine treatment methods demand continued attention from clinicians and researchers alike; the topics outlined above are the purposes of this collection of studies.

## 3. Special Issue Main Accepted Articles

At the time of the writing of this Editorial, there were 15 unique manuscripts accepted for publication in the Special Issue: *Spine Rehabilitation in 2022 and Beyond*. These manuscripts include the following categories of articles: a cross-sectional survey comparing two distinct quality of life questionnaires in adults with scoliosis [46]; a retrospective consecutive cohort investigation examining the relationship of vertebral y-axis rotation of the lumbar spine in functional scoliosis with leg length inequality to sacral shelf lateral tilt angles [47]; a profession wide survey of the chiropractic profession regarding spine radiography utilization examining clinical opinions and experience [48]; a novel clinical manual method comparing manual palpation and motion vs. diagnostic imaging to determine pathological rotational instability movement of the upper cervical spine [49]; four case–control investigations seeking to identify any correlations between spine and posture displacements and patient pain, disability, neurophysiology, and sensory–motor control variables [50,51,52,53]; one case series looking at the relationship between non-surgical sagittal plane cervical spine correction and the improvement in upper cervical spine rotational instability [54]; five randomized trials examining the relationship between correction/reduction of cervical and thoracic posture deformities and spine displacements and improvements of a variety of clinical outcome measures including pain, disability, neurophysiology, range of motion, and sensory–motor control measures [55,56,57,58,59]; and, lastly, one systematic literature review that sought to understand the differences in low back pain and disability characteristics in adults with and without scoliotic spine deformities [60].

Importantly, each one of these 15 accepted manuscripts offers unique and succinct relevant data that provide further evidence that the ‘bio’ (biology and biomechanics) component of the ‘bio-psycho-social’ model of spine care is extremely important to understanding patient pain, disability, and dysfunction and to providing enhanced treatment procedures that improve the outcomes of patient care [46,47,48,49,50,51,52,53,54,55,56,57,58,59,60]. As such, this Special Issue on spine rehabilitation provides useful, cutting-edge, relevant information that should prove to be useful to improve patient care and outcomes in populations suffering from a wide variety of spine related disorders. We thank all the authors of each of these manuscripts for their work, dedication, and insights they provided to bring their team’s data together in an effective scientific manner. We are confident that each of the manuscripts contained in this collection will be well cited and used by future clinicians from many disciplines and researchers to treat patients around the globe and to improve upon the information presented [46,47,48,49,50,51,52,53,54,55,56,57,58,59,60].

## 4. Conclusions

Good quality data currently exist and continue to evolve to support the ‘bio’ element in the biopsychosocial model of chronic pain disorders. This Special Issue, dedicated to ‘spine rehabilitation in 2022’, features highlights of several research avenues taking place, such as the link between altered posture and physical performance, altered posture and pathological conditions, as well as the therapeutic improvement in spine alignment and posture correlating with positive patient outcomes. These lines of research are desperately needed and, unfortunately, continue to be underrecognized. We believe a tidal wave of ‘bio’ evidence is mounting, and a better of the understanding of the biomechanics in spine care will lead to more effective treatments. We hope for the biomechanical spine literature to continue to gain a wider acknowledgement and acceptance by the chronic spine pain community.

## References

[1] Cohen S.P. (2015). Epidemiology, diagnosis, and treatment of neck pain. Mayo Clin. Proc..

[2] Cohen S.P., Hooten W.M. (2017). Advances in the diagnosis and management of neck pain. BMJ.

[3] Kondo Y., Ota R., Fujita H., Miki T., Watanabe Y., Takebayashi T. (2023). Quality of Japanese Online Information on Causes of Neck Pain: A Biopsychosocial Analysis. Cureus.

[4] Chou R., Côté P., Randhawa K., Torres P., Yu H., Nordin M., Hurwitz E.L., Haldeman S., Cedraschi C. (2018). The Global Spine Care Initiative: Applying evidence-based guidelines on the non-invasive management of back and neck pain to low- and middle-income communities. Eur. Spine J..

[5] Liew B.X.W., Hartvigsen J., Scutari M., Kongsted A. (2023). Data-driven network analysis identified subgroup-specific low back pain pathways: A cross-sectional GLA:D Back study. J. Clin. Epidemiol..

[6] Buchbinder R., van Tulder M., Öberg B., Costa L.M., Woolf A., Schoene M., Croft P., Lancet Low Back Pain Series Working Group (2018). Low back pain: A call for action. Lancet.

[7] Martinez-Calderon J., Flores-Cortes M., Morales-Asencio J.M., Luque-Suarez A. (2019). Pain-related fear, pain intensity and function in individuals with chronic musculoskeletal pain: A systematic review and meta-analysis. J. Pain.

[8] Martinez-Calderon J., Jensen M.P., Morales-Asencio J.M., Luque-Suarez A. (2019). Pain Catastrophizing and Function In Individuals With Chronic Musculoskeletal Pain: A Systematic Review and Meta-Analysis. Clin. J. Pain.

[9] Luque-Suarez A., Falla D., Morales-Asencio J.M., Martinez-Calderon J. (2020). Is kinesiophobia and pain catastrophising at baseline associated with chronic pain and disability in whiplash-associated disorders? A systematic review. Br. J. Sports Med..

[10] Oxland T.R. (2016). Fundamental biomechanics of the spine—What we have learned in the past 25 years and future directions. J. Biomech..

[11] Patwardhan A.G., Khayatzadeh S., Havey R.M., Voronov L.I., Smith Z.A., Kalmanson O., Ghanayem A.J., Sears W. (2018). Cervical sagittal balance: A biomechanical perspective can help clinical practice. Eur. Spine J..

[12] Brinjikji W., Diehn F.E., Jarvik J.G., Carr C.M., Kallmes D.F., Murad M.H., Luetmer P.H. (2015). MRI Findings of Disc Degeneration are More Prevalent in Adults with Low Back Pain than in Asymptomatic Controls: A Systematic Review and Meta-Analysis. AJNR Am. J. Neuroradiol..

[13] Raastad J., Reiman M., Coeytaux R., Ledbetter L., Goode A.P. (2015). The association between lumbar spine radiographic features and low back pain: A systematic review and meta-analysis. Semin. Arthritis Rheum..

[14] Din R.U., Cheng X., Yang H. (2022). Diagnostic Role of Magnetic Resonance Imaging in Low Back Pain Caused by Vertebral Endplate Degeneration. J. Magn. Reson. Imaging.

[15] Jamaludin A., Kadir T., Zisserman A., McCall I., Williams F.M.K., Lang H., Buchanan E., Urban J.P.G., Fairbank J.C.T. (2023). ISSLS PRIZE in Clinical Science 2023: Comparison of degenerative MRI features of the intervertebral disc between those with and without chronic low back pain. An exploratory study of two large female populations using automated annotation. Eur. Spine J..

[16] Christensen S.T., Hartvigsen J. (2008). Spinal curves and health: A systematic critical review of the epidemiological literature dealing with associations between sagittal spinal curves and health. J. Manip. Physiol. Ther..

[17] Jenkins H.J., Downie A.S., Moore C.S., French S.D. (2018). Current evidence for spinal X-ray use in the chiropractic profession: A narrative review. Chiropr. Man. Therap..

[18] Corso M., Cancelliere C., Mior S., Kumar V., Smith A., Côté P. (2020). The clinical utility of routine spinal radiographs by chiropractors: A rapid review of the literature. Chiropr. Man. Therap..

[19] Oakley P.A., Betz J.W., Harrison D.E., Siskin L.A., Hirsh D.W., International Chiropractors Association Rapid Response Research Review Subcommittee (2021). Smoke Screen to Distract from Flawed Science: A Response to Côté et al. Over Criticisms to Their Deficient ‘Rapid Review’ on Chiropractic X-Ray Utility. Dose Response.

[20] Oakley P.A., Cuttler J.M., Harrison D.E. (2018). Response to Letters from Anderson and Kawchuk et al.: X-Ray Imaging Is Essential for Contemporary Chiropractic and Manual Therapy Spinal Rehabilitation: Radiography Increases Benefits and Reduces Risks. Dose Response.

[21] Harrison D.E., Betz J., Ferrantelli J.F. (2009). Sagittal spinal curves and health. J. Vertebr. Sublux. Res..

[22] Oakley P.A., Harrison D.E. (2019). Selective usage of medical practice data, misrepresentations, and omission of conflicting data to support the ‘red flag only’ agenda for chiropractic radiography guidelines: A critical assessment of the Jenkins et al. article: “Current evidence for spinal X-ray use in the chiropractic profession”. Ann. Vert. Sublux. Res..

[23] Oakley P.A., Harrison D.E. (2020). Are Restrictive Medical Radiation Imaging Campaigns Misguided? It Seems So: A Case Example of the American Chiropractic Association’s Adoption of “Choosing Wisely”. Dose Response.

[24] Oakley P.A., Betz J.W., Harrison D.E., Siskin L.A., Hirsh D.W., International Chiropractors Association Rapid Response Research Review Subcommittee (2021). Radiophobia Overreaction: College of Chiropractors of British Columbia Revoke Full X-Ray Rights Based on Flawed Study and Radiation Fear-Mongering. Dose Response.

[25] Mekhael E., El Rachkidi R., Saliby R.M., Nassim N., Semaan K., Massaad A., Karam M., Saade M., Ayoub E., Rteil A. (2023). Functional assessment using 3D movement analysis can better predict health-related quality of life outcomes in patients with adult spinal deformity: A machine learning approach. Front. Surg..

[26] Terran J., Schwab F., Shaffrey C.I., Smith J.S., Devos P., Ames C.P., Fu K.M., Burton D., Hostin R., Klineberg E. (2013). The SRS-Schwab adult spinal deformity classification: Assessment and clinical correlations based on a prospective operative and nonoperative cohort. Neurosurgery.

[27] Pellisé F., Vila-Casademunt A., Ferrer M., Domingo-Sàbat M., Bagó J., Pérez-Grueso F.J., Alanay A., Mannion A.F., Acaroglu E., European Spine Study Group (ESSG) (2015). Impact on health related quality of life of adult spinal deformity (ASD) compared with other chronic conditions. Eur. Spine J..

[28] Kyrölä K., Repo J., Mecklin J.P., Ylinen J., Kautiainen H., Häkkinen A. (2018). Spinopelvic Changes Based on the Simplified SRS-Schwab Adult Spinal Deformity Classification: Relationships with Disability and Health-Related Quality of Life in Adult Patients With Prolonged Degenerative Spinal Disorders. Spine.

[29] Kim H.J., Yang J.H., Chang D.G., Lenke L.G., Suh S.W., Nam Y., Park S.C., Suk S.I. (2022). Adult Spinal Deformity: A Comprehensive Review of Current Advances and Future Directions. Asian Spine J..

[30] Yahata M., Watanabe K., Tashi H., Ohashi M., Yoda T., Nawata A., Nakamura K., Kawashima H. (2023). Impact of spinal sagittal malalignment on locomotive syndrome and physical function in community-dwelling middle aged and older women. BMC Musculoskelet. Disord..

[31] Chun S.W., Lim C.Y., Kim K., Hwang J., Chung S.G. (2017). The relationships between low back pain and lumbar lordosis: A systematic review and meta-analysis. Spine J..

[32] Sadler S.G., Spink M.J., Ho A., De Jonge X.J., Chuter V.H. (2017). Restriction in lateral bending range of motion, lumbar lordosis, and hamstring flexibility predicts the development of low back pain: A systematic review of prospective cohort studies. BMC Musculoskelet. Disord..

[33] Roghani T., Allen D.D., Gladin A., Rahimi A., Mehrabi M., Rezaeian Z.S., Farajzadegan Z., Katzman W.B. (2023). The Association Between Physical Function and Hyperkyphosis in Older Females: A Systematic Review and Meta-analysis. J. Geriatr. Phys. Ther..

[34] Petcharaporn M., Pawelek J., Bastrom T., Lonner B., Newton P.O. (2007). The relationship between thoracic hyperkyphosis and the Scoliosis Research Society outcomes instrument. Spine.

[35] Garrido E., Roberts S.B., Duckworth A., Fournier J. (2021). Long-term follow-up of untreated Scheuermann’s kyphosis. Spine Deform..

[36] Ling F.P., Chevillotte T., Leglise A., Thompson W., Bouthors C., Le Huec J.C. (2018). Which parameters are relevant in sagittal balance analysis of the cervical spine? A literature review. Eur. Spine J..

[37] Protopsaltis T.S., Scheer J.K., Terran J.S., Smith J.S., Hamilton D.K., Kim H.J., Mundis GMJr Hart R.A., McCarthy I.M., Klineberg E., Lafage V. (2015). How the neck affects the back: Changes in regional cervical sagittal alignment correlate to HRQOL improvement in adult thoracolumbar deformity patients at 2-year follow-up. J. Neurosurg. Spine.

[38] Oakley P.A., Ehsani N.N., Moustafa I.M., Harrison D.E. (2020). Restoring lumbar lordosis: A systematic review of controlled trials utilizing Chiropractic Bio Physics^®^ (CBP^®^) non-surgical approach to increasing lumbar lordosis in the treatment of low back disorders. J. Phys. Ther. Sci..

[39] Oakley P.A., Ehsani N.N., Moustafa I.M., Harrison D.E. (2021). Restoring cervical lordosis by cervical extension traction methods in the treatment of cervical spine disorders: A systematic review of controlled trials. J. Phys. Ther. Sci..

[40] Oakley P.A., Harrison D.E. (2018). Reducing Thoracic Hyperkyphosis Subluxation Deformity: A Systematic Review of Chiropractic BioPhysics^®^ Methods Employed in its Structural Improvement. J. Contemp. Chiropr..

[41] Jenkins H.J., Downie A.S., Fernandez M., Hancock M.J. (2021). Decreasing thoracic hyperkyphosis—Which treatments are most effective? A systematic literature review and meta-analysis. Musculoskelet. Sci. Pract..

[42] Ponzano M., Tibert N., Bansal S., Katzman W., Giangregorio L. (2021). Exercise for improving age-related hyperkyphosis: A systematic review and meta-analysis with GRADE assessment. Arch. Osteoporos..

[43] Harrison D.E., Oakley P.A., Bernardo-Filho M. (2022). An introduction to Chiropractic BioPhysics^®^ (CBP^®^) technique: A full spine rehabilitation approach to reducing spine deformities. Complementary Therapies.

[44] Oakley P.A., Moustafa I.M., Harrison D.E., Bettany-Saltikov J. (2019). Restoration of cervical and lumbar lordosis: CBP^®^ methods overview. Spinal Deformities in Adolescents, Adults and Older Adults.

[45] Oakley P.A., Moustafa I.M., Harrison D.E., Bernardo-Filho M. (2021). The Influence of Sagittal Plane Spine Alignment on Neurophysiology and Sensorimotor Control Measures: Optimization of Function through Structural Correction. Therapy Approaches in Neurological Disorders.

[46] Zaina F., Ferrario I., Caronni A., Scarano S., Donzelli S., Negrini S. (2023). Measuring Quality of Life in Adults with Scoliosis: A Cross-Sectional Study Comparing SRS-22 and ISYQOL Questionnaires. J. Clin. Med..

[47] Marsiolo M., Careri S., Bandinelli D., Toniolo R.M., Aulisa A.G. (2023). Vertebral rotation in functional scoliosis caused by limb-length inequality: Correlation between rotation, limb length inequality, and obliquity of the sacral shelf. J. Clin. Med..

[48] Arnone P.A., Kraus S.J., Farmen D., Lightstone D.F., Jaeger J., Theodossis C. (2023). Examining Clinical Opinion and Experience Regarding Utilization of Plain Radiography of the Spine: Evidence from Surveying the Chiropractic Profession. J. Clin. Med..

[49] Kaale B.R., McArthur T.J., Barbosa M.H., Freeman M.D. (2023). Post-Traumatic Atlanto-Axial Instability: A Combined Clinical and Radiological Approach for the Diagnosis of Pathological Rotational Movement in the Upper Cervical Spine. J. Clin. Med..

[50] Moustafa I.M., Shousha T., Arumugam A., Harrison D.E. (2023). Is Thoracic Kyphosis Relevant to Pain, Autonomic Nervous System Function, Disability, and Cervical Sensorimotor Control in Patients with Chronic Nonspecific Neck Pain?. J. Clin. Med..

[51] Moustafa I.M., Diab A.A.M., Harrison D.E. (2023). Does Forward Head Posture Influence Somatosensory Evoked Potentials and Somatosensory Processing in Asymptomatic Young Adults?. J. Clin. Med..

[52] Kamel M., Moustafa I.M., Kim M., Oakley P.A., Harrison D.E. (2023). Alterations in Cervical Nerve Root Function during Different Sitting Positions in Adults with and without Forward Head Posture: A Cross-Sectional Study. J. Clin. Med..

[53] Ahbouch A., Moustafa I.M., Shousha T., Arumugam A., Oakley P., Harrison D.E. (2023). An Investigation of the Association between 3D Spinal Alignment and Fibromyalgia. J. Clin. Med..

[54] Katz E.A., Katz S.B., Freeman M.D. (2023). Non-Surgical Management of Upper Cervical Instability via Improved Cervical Lordosis: A Case Series of Adult Patients. J. Clin. Med..

[55] Suwaidi A.S.A., Moustafa I.M., Kim M., Oakley P.A., Harrison D.E. (2023). A Comparison of Two Forward Head Posture Corrective Approaches in Elderly with Chronic Non-Specific Neck Pain: A Randomized Controlled Study. J. Clin. Med..

[56] Youssef A.S.A., Moustafa I.M., El Melhat A.M., Huang X., Oakley P.A., Harrison D.E. (2022). Randomized Feasibility Pilot Trial of Adding a New Three-Dimensional Adjustable Posture-Corrective Orthotic to a Multi-Modal Program for the Treatment of Nonspecific Neck Pain. J. Clin. Med..

[57] Moustafa I.M., Diab A.A., Harrison D.E. (2022). The Efficacy of Cervical Lordosis Rehabilitation for Nerve Root Function and Pain in Cervical Spondylotic Radiculopathy: A Randomized Trial with 2-Year Follow-Up. J. Clin. Med..

[58] Moustafa I.M., Shousha T.M., Walton L.M., Raigangar V., Harrison D.E. (2022). Reduction of Thoracic Hyper-Kyphosis Improves Short and Long Term Outcomes in Patients with Chronic Nonspecific Neck Pain: A Randomized Controlled Trial. J. Clin. Med..

[59] Moustafa I.M., Diab A.A.M., Harrison D.E. (2022). Does Improvement towards a Normal Cervical Sagittal Configuration Aid in the Management of Lumbosacral Radiculopathy: A Randomized Controlled Trial. J. Clin. Med..

[60] Zaina F., Marchese R., Donzelli S., Cordani C., Pulici C., McAviney J., Negrini S. (2023). Current Knowledge on the Different Characteristics of Back Pain in Adults with and without Scoliosis: A Systematic Review. J. Clin. Med..

